# Discovering Cathodic Biocompatibility for Aqueous Zn–MnO_2_ Battery: An Integrating Biomass Carbon Strategy

**DOI:** 10.1007/s40820-024-01334-3

**Published:** 2024-02-05

**Authors:** Wei Lv, Zilei Shen, Xudong Li, Jingwen Meng, Weijie Yang, Fang Ding, Xing Ju, Feng Ye, Yiming Li, Xuefeng Lyu, Miaomiao Wang, Yonglan Tian, Chao Xu

**Affiliations:** 1 Institute of Energy Power Innovation, North China Electric Power University, Beijing, 102206 People’s Republic of China; 2 Department of Power Engineering, School of Energy, Power and Mechanical Engineering, North China Electric Power University, Baoding, 071003 People’s Republic of China; 3 Key Laboratory of RNA Biology, Institute of Biophysics, Chinese Academy of Sciences, Beijing, 100101 People’s Republic of China; 4 Collaborative Innovation Center of Integrated Exploitation of Bayan Obo Multi-Metal Resources, Inner Mongolia University of Science and Technology, Baotou, 014010 People’s Republic of China

**Keywords:** Aqueous Zn-ion batteries, Biocompatibility, Jahn–Teller effect, Mn domains, γ-MnO_2_

## Abstract

Developing high-performance aqueous Zn-ion batteries from sustainable biomass becomes increasingly vital for large-scale energy storage in the foreseeable future. Therefore, γ-MnO_2_ uniformly loaded on N-doped carbon derived from grapefruit peel is successfully fabricated in this work, and particularly the composite cathode with carbon carrier quality percentage of 20 wt% delivers the specific capacity of 391.2 mAh g^−1^ at 0.1 A g^−1^, outstanding cyclic stability of 92.17% after 3000 cycles at 5 A g^−1^, and remarkable energy density of 553.12 Wh kg^−1^ together with superior coulombic efficiency of ~ 100%. Additionally, the cathodic biosafety is further explored specifically through in vitro cell toxicity experiments, which verifies its tremendous potential in the application of clinical medicine. Besides, Zinc ion energy storage mechanism of the cathode is mainly discussed from the aspects of Jahn–Teller effect and Mn domains distribution combined with theoretical analysis and experimental data. Thus, a novel perspective of the conversion from biomass waste to biocompatible Mn-based cathode is successfully developed.

**Supplementary Information:**

The online version contains supplementary material available at 10.1007/s40820-024-01334-3.

## Highlights

- γ-MnO_2_ loaded on N-doped biomass carbon from grapefruit peel is firstly developed.
- The splendid cathodic properties (e.g., coulombic efficiency: ~ 100%, energy density: 553.12 Wh kg^−1^) are gained.
- The biomass strategy guaranteed via cytotoxicity test shows a clinical potential.
- Zn-ion storage efficiency is boosted mainly by regulating Mn–O bond and Mn domains.

**Supplementary Information:**

The online version contains supplementary material available at 10.1007/s40820-024-01334-3.

## Graphical abstract

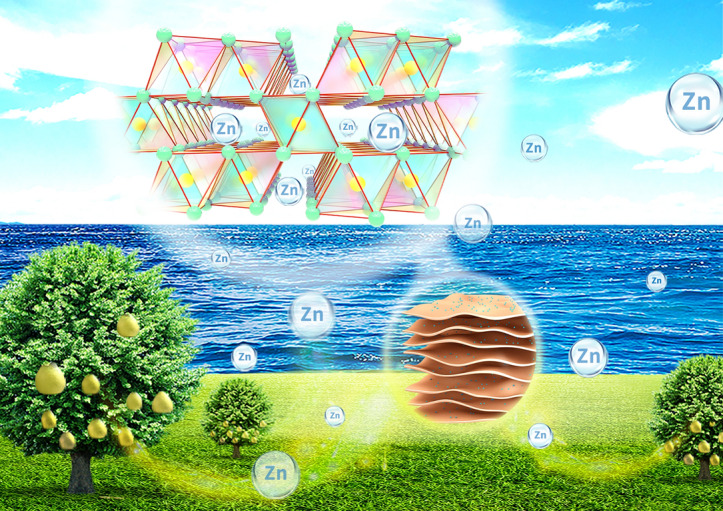

## Introduction

With the continuous global carbon emission, biomass energy as a kind of green renewable, biodegradable and nontoxic energy resource has attracted considerable attention [1–8]. In other words, the recycling of biomass resources as a feasible way to achieve carbon neutrality shows tremendous potential in the field of energy [9–16], materials, health care, and so on [17–24]. In particular, the development of high safe and green large-scale energy storage technology using biomass is of great significance for building a clean and low-carbon modern energy system [25–32]. Lithium-ion batteries have been widely used in new energy electric vehicles [33–46], but the utilization of flammable organic electrolytes plus high manufacturing costs for lithium batteries are not conducive to the application in large-scale energy storage [47–57]. Therefore, the inexpensive and eco-friendly aqueous Zn-ion batteries (AZIBs) with high-safety are considered to have great potential in massive-scale energy storage [58–65]. There are many factors affecting the properties of AZIBs, among which the development of stable cathodic materials becomes the key [66–73], and thereinto, MnO_2_ with the characteristics of diverse structures (α-, β-, γ-, and δ-, etc.), low price, and environmental friendliness has been widely used as cathodic materials of AZIBs [74–81]. Nevertheless, the repeated insertion/extraction of Zn^2+^ during charge–discharge process leads to the structural deformation for MnO_2_ [82–88]. Meanwhile, Mn^4+^ is prone to be reduced to Mn^3+^ so as to induce the Jahn–Teller effect and lattice distortion [89, 90]. In addition, the disproportionation reaction of Mn^3+^ also promotes the dissolution of MnO_2_ [91–96]. Thus, exploring sustainable and renewable biomass resources to improve the structural stability of MnO_2_ would be a “Win–Win” strategy.

The recombination of carbon materials and MnO_2_ has been identified as an important way to optimize the cathodic performance of aqueous Zn–MnO_2_ batteries [97–99]. Chen et al. synthesized carbon nanofiber@δ-MnO_2_ with a facile method combining solid-grinding and wet-chemical reaction, and achieved a capacity of 277 mAh g^−1^ and capacity retention of 79.78% after 700 cycles at 200 mA g^−1^ [100]. Li et al. prepared N-doped carbon nanowires incorporated with δ-MnO_2_ by hydrothermal method, the discharge capacities of which were 325 mAh g^−1^ at 100 mA g^−1^ and 90 mAh g^−1^ at 2 A g^−1^ respectively, and its cycle life after 2500 cycles was 95% at 2 A g^−1^ [101]. Huang et al. electrochemically deposited α-MnO_2_ onto carbon nanotube as cathode, which achieved the specific capacities of 292.7 and 105.6 mAh g^−1^ at 0.2 and 3 mA cm^−2^ respectively, and its cycling life remained 88.5% after 300 cycles at 0.3 mA cm^−2^ [102]. Moreover, Kim et al. reported a carbon-coated α-MnO_2_ cathode, which exhibits a discharge capacity of 272 mAh g^−1^ and cycle life of 69.49% after 50 cycles at 66 mA g^−1^ [103]. Overall, carbon materials used to be compound with MnO_2_ are mainly based on the conventional commercial materials such as carbon nanofiber, carbon nanotube, etc. Therefore, the research on how to improve Zn-ion storage performance by constructing Mn-based cathode compounded with carbon materials derived from biomass, especially the inexpensive and renewable biomass waste, is still rare.

In this work, the abundant grapefruit peel as a natural biomass carbon source is adopted to synthesize N-doped carbon carrier powder (CP) through a simple calcination in N_2_ atmosphere, and γ-MnO_2_ prepared by electro-deposition is uniformly loaded onto CP to obtain the composite cathode (γ-MnO_2_@CP). A systematic study about the improving mechanism of Zn-ion storage efficiency via compounding CP is conducted from multiple perspectives of Jahn–Teller effect, Mn valence, and Mn domains, etc. Besides, the in vitro cytotoxicity experiments of pure γ-MnO_2_ and γ-MnO_2_@CP are carried out to investigate the application prospect in the field of biomedicine. Therefore, the above research provides a valuable guidance for the comprehensive utilization of wasted biomass to design high-performance MnO_2_-biomass carbon cathode.

## Experimental Section

### Sample Preparation

The grapefruit peel was washed three times by deionized water, then heated at 80 °C for 3 h and crushed into yellow powder, which was heated at 650 °C for 2 h under a nitrogen atmosphere to obtain CP. A three-electrode system composed of a stainless steel working electrode, a saturated calomel reference electrode, and a platinum plate counter electrode was used for MnO_2_ electro-deposition under the current density of 5 mA cm^−2^ for 30 min with electro-magnetic stirring, and the electrolyte was made up of 0.5 M Mn(CH_3_COO)_2_ and 0.5 M Na_2_SO_4_, the chemical reaction occurred on the surface of stainless steel working electrode is described as: Mn^2+^  + 2H_2_O → MnO_2_ + 4H^+^  + 2e^−^. Finally, the thin films of MnO_2_ electrochemically deposited on the stainless steel electrode were scrapped off. The mixtures of as-prepared MnO_2_ and CP (Quality percentage of CP: 10, 20, 30, and 40 wt%) were magnetically stirred in deionized water for 1 h, and then ultrasonically dispersed for another 1 h respectively, the above solutions were centrifuged and then dried at 80 °C (Heating rate: ~ 2 °C min^−1^) in air for 3 h to obtain the cathodic active materials, which were labelled as CP-10, CP-20, CP-30 and CP-40, respectively. Besides, the as-prepared MnO_2_ mentioned above was labelled as CP-0.

### Materials Characterization

The X-ray diffraction (XRD) profiles were measured with Bruker-D8 Advance X-ray diffractometer (Cu Kα radiation, 2θ step: 0.02°) and analyzed with Jade 5.0 software. The spectroscopic property was tested though PerkinElmer Spectrum 100 FTIR. The micro morphology was observed through JEOL JEM-2100F TEM and Nova Nano SEM 450. The composition and valence state were tested by Thermo ESCALAB 250XI XPS.

### Computational Calculation

All the theoretical calculation about the models of Zn^2+^ intercalating into the tunnel structures of pure γ-MnO_2_ and γ-MnO_2_@CP were performed with VASP software based on density functional theory (DFT) in this work. The migration behavior of Zn^2+^ was computed by mean square displacement (*MSD*) using the equation $MSD { = }\frac{{1}}{{M{ }}}\sum\nolimits_{{i{ = 1}}}^{M} { \left| { r_{i } \left( t \right) - r_{i} \left( {0} \right) } \right|^{{2}} }$, where *r*_i_ (0) is the initial position of Zn^2+^, *r*_i_ (*t*) is the terminal position of Zn^2+^, and *M* is the number of simulation, then the diffusion coefficient (*D*) of Zn^2+^ was calculated by fitting 6*t* to *MSD* as the Einstein relation: $D = \mathop {\lim }\limits_{t \to \infty } \frac{MSD}{{6t}}$ [74]. The adsorption energy (*E*_ads_) values of Zn^2+^ and OH^−^ on the tunnel structural surface of γ-MnO_2_ were calculated, and the bader charges of the above two models were also simulated respectively. Meanwhile, Mn–O bond lengths of MnO_6_ octahedron in the above two models were calculated for structural analysis.

### Electrochemical Properties

The cathodic active materials (CP-0, CP-10, CP-20, CP-30, and CP-40), acetylene black and poly-vinylidene fluoride were mixed together in accordance with the gravimetric ratio of 7:2:1, and N-methyl-2-pyrrolidone was added into the above mixtures to produce black slurries, which were then painted on 5 stainless steel webs (Diameter: ~ 14 mm) and then heated at 80 °C (Heating rate: ~ 2 °C min^−1^) in air for 8 h to obtain the cathodic current collectors (Load quality of active substance: ~ 2 mg cm^−2^). A CR2032 button battery configuration with a Whatman glass-fiber diaphragm (Grade GF/D), Zn anode, and the electrolyte of 2 mol L^−1^ ZnSO_4_ and 0.1 mol L^−1^ MnSO_4_ was used to estimate the cathodic active materials, the prepared button batteries shared the same numbers as CP-0, CP-10, CP-20, CP-30, and CP-40 respectively. The galvanostatic charge/discharge performance was tested using LANHE CT3002A equipment (Voltage: 0.8–1.8 V) based on the active material mass. The cyclic voltammetry (CV) curves were recorded using a CHI660E electrochemical work station (Scan rate: 0.1, 0.2, 0.4, 0.6, and 0.8 mV s^−1^), then *b* value was obtained according to the equation log(*i*) = log(*a*) + *b*log(*v*) (*i*: Peak current; *v*: Scan rate; *a* and *b*: Adjustable values), the pseudocapacitive fitting was calculated on the basis of the relational expression *i*(*V*) = k_1_*v* + k_2_*v*^0.5^ (k_1_*v*: Non-diffusion controlled contribution; k_2_*v*^0.5^: Diffusion controlled contribution).

### In Vitro Cytotoxicity

3T3 mouse embryonic fibroblast cells were cultured in DMEM medium (Gibco, USA) supplemented with 10% fetal bovine serum (FBS, Gibco, USA) and 1% penicillin/streptomycin (Gibco, USA) at 37 °C. In vitro cell viability was evaluated by CCK-8 assay against 3T3 cells. Briefly, cells were seeded in 96-well plates and incubated with the as-synthesized materials, including CP-0, CP-10, CP-20, CP-30, and CP-40. 10 μL CCK-8 solution was added to each well and the absorbance was recorded on a microplate reader (EnSpire, USA) at 490 nm. Cell apoptosis assay was conducted by using an Annexin V-FITC/PI apoptosis detection kit (Beyotime, China). For apoptosis assay, 3T3 cells were seeded into 6-well plates and incubated with the above-mentioned materials for 48 h. Thereafter, cells were stained with Annexin V FITC/PI for 30 min and the apoptosis percentage was detected by a flow cytometer (BD FACS Calibur, USA). Calcein AM/PI Co-staining was performed to detect the live/dead cells under different treatments. Following incubation cells were stained with calcein AM and PI for 30 min, and the stained cells were imaged by a fluorescence microscope (NIKON, Japan). For the above experiments, cells in PBS were set as the control group.

## Results and Discussions

### Microstructure

**Fig. 1:**
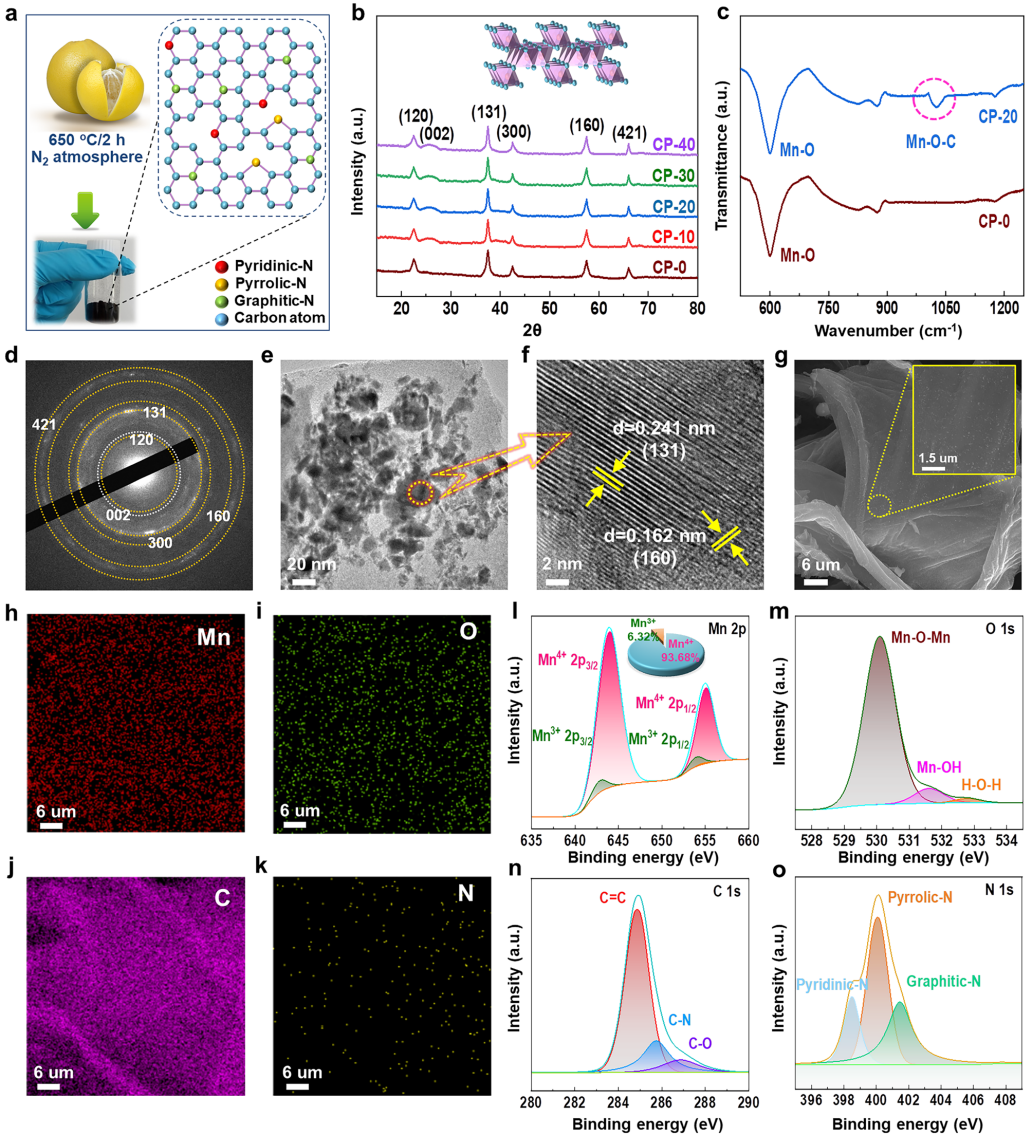
** a** Schematic diagram for the preparation of CP. **b** XRD patterns of CP-0, CP-10, CP-20, CP-30, and CP-40. **c** FTIR spectra of CP-0 and CP-20. **d** SAED, **e** and **f** HRTEM, **g** SEM, **h–k** EDS of CP-20. XPS high-resolution patterns of **l** Mn 2*p*, **m** O 1*s*, **n** C 1*s*, and **o** N 1*s* of CP-20.

The preparation detail of CP is schematically illustrated in Fig. 1a, and the N-doped biomass carbon is expected to be obtained. As shown in Fig. 1b, all the samples display five characteristic peaks indexed to (120), (131), (300), (160), and (421) crystal planes, which means that γ-MnO_2_ is successfully synthesized by electro-deposition corresponding to JCPDS No. 14-0644 [104], and the broad peaks at around 26° belonging to (002) crystal plane of carbon for CP-10, CP-20, CP-30, and CP-40 are also observed [100]. Compared with CP-0, a new peak at around 1025 cm^−1^ corresponding to Mn–O–C bond is observed in the FTIR spectrum of CP-20 (Fig. 1c), which once again demonstrates the successful recombination of CP and γ-MnO_2_ for CP-20. The characteristic planes of CP and γ-MnO_2_ are also observed in SAED pattern (Fig. 1d), and the HRTEM images of CP-20 indicate that the interplanar spacings of 0.162 and 0.241 nm are well indexed to the lattice planes of (160) and (131) for γ-MnO_2_ (Fig. 1e, f), which further confirms the accuracy of the analytical result from Fig. 1b, d. Figure 1g shows a successful loading of γ-MnO_2_ nanoparticles on the surface of irregular flower-like CP. The EDS result shows a uniform element distribution of Mn, O, C, and N for CP-20 (Fig. 1h–k), and the XPS result once again affirms the coexistence of Mn, O, C, and N on the surface of CP-20 (Fig. 1l–o). Besides, Fig. S1 certifies the existence of Mn and O elements for CP-0, and Figs. S2, S3, and S4 also reveal the existence of Mn, O, C, and N elements for CP-10, CP-30, and CP-40 respectively, meanwhile the coexistence of Mn^3+^ and Mn^4+^ is revealed for all the samples.

The Jahn–Teller effect primarily due to high-spin Mn^3+^ with (t_2g_)^3^(e_g_)^1^ electron configuration in 3*d* orbital usually leads to detrimental structural disorder for MnO_2_, so MnO_2_ with a higher Mn valence is promising to exhibit excellent long-term cycling stability [105]. Therefore, the compound strategy of γ-MnO_2_ with CP is beneficial to reduce the content of Mn^3+^, and it's worth noting that CP-20 exhibits the highest percent of Mn^4+^ (93.68%) and the lowest percent of Mn^3+^ (6.32%), which foreshadows the weakest Jahn–Teller effect in CP-20 (Figs. 1l and S1a, S2a, S3a, S4a).

### Theoretical Calculation

**Fig. 2:**
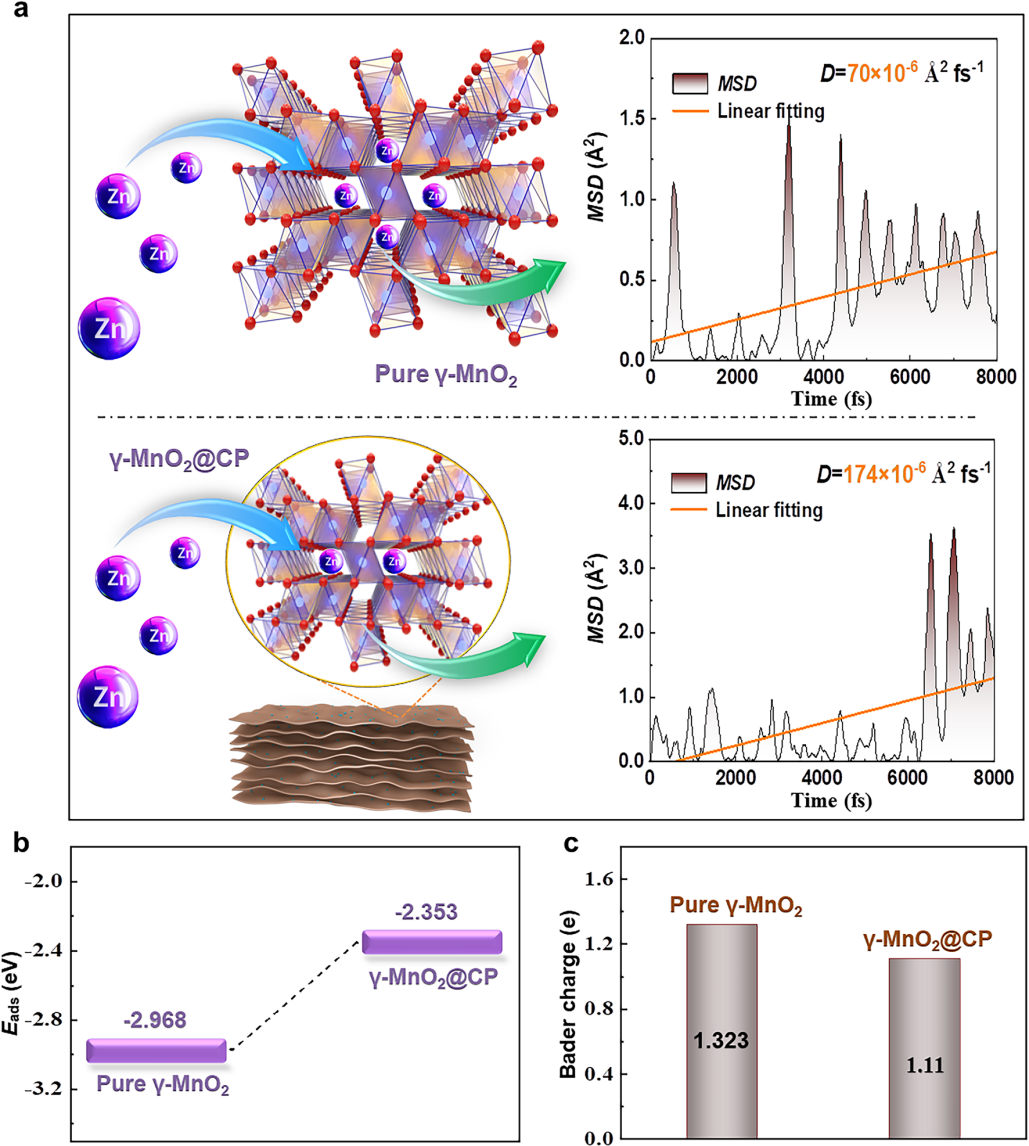
Theoretical calculated results of **a** *MSD*, **b** *E*_ads_, and **c** bader charge about the models of Zn^2+^ intercalating into the tunnel structures of pure γ-MnO_2_ and γ-MnO_2_@CP

As shown in Fig. 2a, the calculated *D* values of Zn^2+^ in the internal structures of pure γ-MnO_2_ and γ-MnO_2_@CP are 70 × 10^–6^ and 174 × 10^–6^ Å^2^ fs^−1^ respectively, and it can be seen that the CP composite strategy effectively improves Zn^2+^ kinetics. The *E*_ads_ values of Zn^2+^ on the tunnel-shaped surface of pure γ-MnO_2_ and γ-MnO_2_@CP are − 2.968 and − 2.353 eV respectively, which demonstrates that Zn^2+^ is more liable to migrate smoothly inside γ-MnO_2_@CP (Fig. 2b). Bader charge (1.11*e*) of Zn^2+^ and γ-MnO_2_@CP group is less than that (1.323*e*) of Zn^2+^ and pure γ-MnO_2_ group, and this implies a more obvious electron transfer tendency and a stronger binding interaction between Zn^2+^ and pure γ-MnO_2_, which hinders the diffusion of Zn^2+^ (Fig. 2c). Besides, the nano-sized Mn-based cathode effectively reduces the migration time of Zn^2+^ according to the equation *τ*_eq_ = *L*^2^/2*D* (*τ*_eq_: Diffusion time; *L*: Material size; *D*: Diffusion coefficient) [69], so the combination of nanocrystallization for γ-MnO_2_ (Fig. 1g) and CP composite strategy would be helpful to boost Zn storage efficiency theoretically.

### Electrochemical Property

**Fig. 3:**
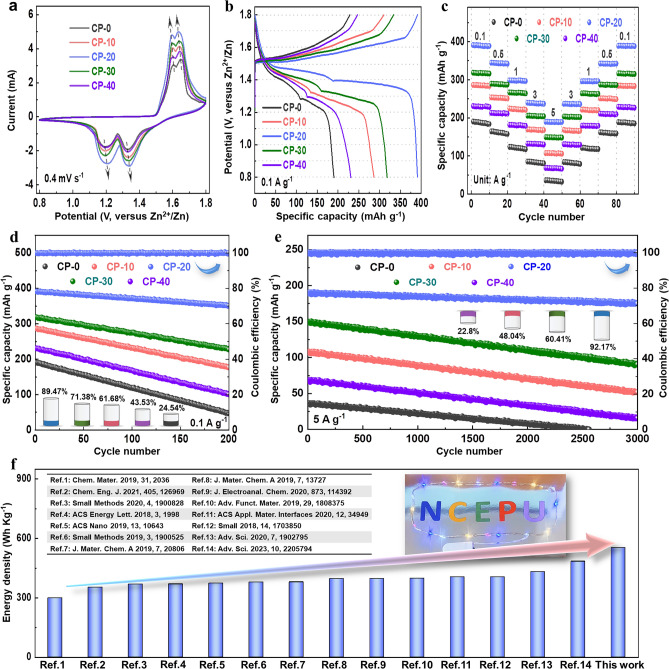
Electrochemical test results of **a** CV curves, **b** constant current charge–discharge profiles, **c** high rate discharge ability, **d** 200 times cycling performance, and **e** 3000 times cyclic stability of CP-0, CP-10, CP-20, CP-30, and CP-40. **f** Comparison diagram about the energy density calculated based on cathodic active material mass between literatures and CP-20

It is worth noting that the cathodic peaks shift toward higher values and the anodic peaks shift toward lower values for CP-10, CP-20, CP-30, and CP-40 compared with those of CP-0 from the CV curves at 0.4 mV s^−1^, which indicates the reduced inherent voltage polarization plausibly related to CP composite, and especially the smallest polarization is seen in CP-20, and CP-20 also shows the highest peak current density implying its largest electrochemical capacity (Fig. 3a). CP-20 with a medium potential of ~ 1.4 V displays a maximum discharge capacity of 391.2 mA g^−1^ and a minimum voltage hysteresis when the current density is 0.1 A g^−1^ among the samples (Fig. 3b). The high rate discharge ability of γ-MnO_2_ mixed with CP are better than that of pure γ-MnO_2_ when the current density increases from 0.1 to 5 A g^−1^ and then reduces to 0.1 A g^−1^, besides CP-20 also exhibits the optimal discharge capability of 189.8 mAh g^−1^ at 5 A g^−1^ compared with other samples (Fig. 3c). The cyclic stability of CP-0 is only 24.54% after 200 cycling times at 0.1 A g^−1^, while CP-20 demonstrates the best cycling performance (89.47%) and coulombic efficiency (~ 100%) (Fig. 3d). In particular, the 3000 times cycling life of CP-20 achieves even high up to 92.17%, and its coulombic efficiency still maintains at ~ 100% at 5 A g^−1^ (Fig. 3e). Furthermore, the specific energy density of CP-20 at 0.1 A g^−1^ reaches 553.12 Wh kg^−1^, which is superior to that of CP-0 (250.32 Wh kg^−1^), CP-10 (383.08 Wh kg^−1^), CP-30 (427.00 Wh kg^−1^) and CP-40 (307.21 Wh kg^−1^). More importantly, the energy density of CP-20 in our work is also superior to those reported in literatures (Fig. 3f). To sum up, the recombination strategy with CP is of benefit to the electrochemical performance enhancement for γ-MnO_2_.

As shown in Figs. S5a, S6a, S7a, S8a, and S9a, the phenomenon that the cathodic and anodic peaks of CV curves for all the samples shift toward negative and positive potentials respectively as the scan rate increases reveals a distinct insertion/deinsertion energy storage behavior of Zn^2+^ [74]. The CP-20 sample, by contrast, has a stronger redox peak and smaller gap between cathodic and anodic peak, which indicates that CP-20 is promising to have faster dynamics, less polarization and better long cycle performance among the samples. The calculated pseudocapacitive proportion results (Figs. S5b, S6b, S7b, S8b, and S9b) show that CP-20 exhibits the maximum pseudocapacitance contribution, and it is again verified that the reinforced pseudocapacitance behavior by CP composite strategy is beneficial to ameliorate the electrochemical characteristics of γ-MnO_2_. Usually, a larger *b*-value (close to 1.0) is deemed to be advantageous for strengthening pseudocapacitance behavior so as to improve energy storage dynamics, obviously *b*-value of CP-20 is lager than that of other samples according to Figs. S5c, S6c, S7c, S8c, and S9c, and this also reveals the effectively promoted pseudocapacitance behaviour of CP-20. Furthermore, The fact that the peak1 and peak2 offset values of CP-20 are the smallest demonstrates again its enhanced intercalation pseudocapacitance (Figs. S5d, S6d, S7d, S8d, and S9d), which strongly supports the above fitting conclusions about pseudocapacitive and *b*-value. More significantly, the presence of pyridinic-N and pyrrolic-N is considered to be responsible for boosting the pseudocapacitance behavior of electrode materials [106], and this might be another key factor to improve the pseudocapacitance characteristics of γ-MnO_2_ via CP composite. (Figs. 1o and S2d, S3d, S4d).

### Mechanism Analysis

**Fig. 4:**
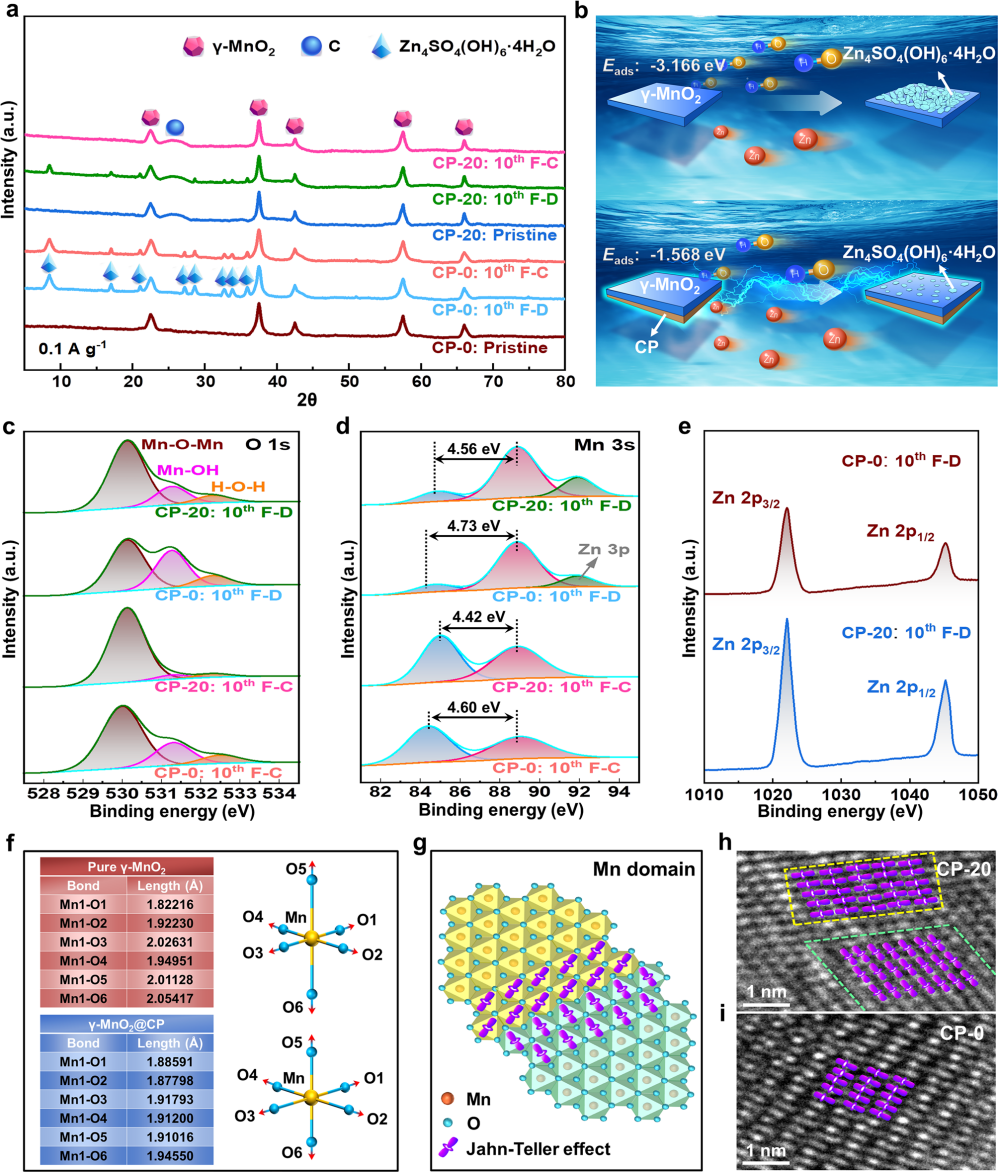
** a** Ex-situ XRD of CP-0 and CP-20 at F-D/F–C states on the 10th cycle. **b** Sketch map at F-D state and the calculated *E*_ads_ values of OH^−^ to the surface of γ-MnO_2_. XPS high-resolution patterns of **c** O 1*s*, **d** Mn 3*s* and **e** Zn 2*p* of CP-0 and CP-20. **f** Calculated Mn–O bonding distances of MnO_6_ octahedra of pure γ-MnO_2_ and γ-MnO_2_@CP. **g** Schematic Mn domains and **h**, **i** actually observed Mn domains

Under the current density of 0.1 A g^−1^, the by-product Zn_4_SO_4_(OH)_6_·4H_2_O (JCPDS No. 44-0673) emerges at the fully discharged (F-D) state and almost completely fades away at the fully charged (F–C) state on the 10th cycle for CP-20, and the reversible reaction is represented by the following equation:$\upgamma - {\text{MnO}}_{2} + {\text{SO}}_{4}^{2 - } + 4{\text{Zn}}^{2 + } + 2{\text{OH}}^{ - } + 6{\text{H}}_{2} {\text{O}} + 2{\text{e}}^{ - } \leftrightarrow {\text{Mn}}^{2 + } + {\text{Zn}}_{4} {\text{SO}}_{4} ({\text{OH}})_{6} \cdot 4{\text{H}}_{2} {\text{O}}$ , while Zn_4_SO_4_(OH)_6_·4H_2_O always exists at F–D and F–C states for CP-0 (Fig. 4a). Noticeably, the calculated *E*_ads_ values of OH^−^ with the surfaces of pure γ-MnO_2_ and γ-MnO_2_@CP are − 3.166 and − 1.568 eV respectively (Fig. 4b), this obvious difference illustrates that OH^−^ is more liable to be adsorbed on the surface of pure γ-MnO_2_ to promote the formation of Zn_4_SO_4_(OH)_6_·4H_2_O, which reconfirms the conclusion from Fig. 4a. Herein, the CP composite strategy effectively inhibits the side effect and ultimately increases the reversibility and efficiency of Zn-ion energy storage.

The O 1* s* XPS spectra of CP-0 and CP-20 are fitted into tetravalent Mn–O–Mn bonds, trivalent Mn–OH bonds, and H–O–H bonds for residual water, correspondingly the average Mn valences of CP-0/CP-20 are estimated to be 3.6 +/3.8 + at F–D state and 3.75 +/3.95 + at F–C state on the 10th cycle according to the area contributions of Mn–O–Mn and Mn–OH components respectively (Fig. 4c). More precisely, the average oxidation state (AOS) of Mn can be computed based on the equation AOS = 8.95–1.13 Δ*E*_Mn3s_, where Δ*E*_Mn3s_ is the energy difference between the main and satellite peaks in Mn 3*s* XPS spectra [107], so the AOS of Mn in CP-0 (F–D), CP-0 (F–C), CP-20 (F–D), and CP-20 (F–C) on the 10th cycle are also 3.6 +, 3.75 +, 3.8 +, and 3.95 + respectively (Fig. 4d), thus the calculated Mn valence from Mn 3*s* and O 1*s* XPS spectra are entirely the same, which further illustrates that the CP composite is beneficial to suppress the Jahn–Teller effect by regulating Mn valence, besides the Zn 3*p* peak of CP-0 is also lower than that of CP-20. Meanwhile, the Zn 2*p* peaks of CP-20 are apparently stronger than that of CP-0 on the 10th cycle at F-D state (Fig. 4e), which reconfirms the conclusion in Fig. 4d. Different from the Mn–O bonds of γ-MnO_2_@CP nearly without distortion, the Jahn–Teller effect induces a geometric distortion with two longer (O5 and O6) Mn–O bonds of pure γ-MnO_2_ according to theoretical calculation (Fig. 4f). The formation of Mn domains is supposed to disrupt the cooperativity of Jahn–Teller effect as schematically shown in Fig. 4g [108], and the Mn domains with different orientation are also visually identified in CP-20 (Fig. 4h) but not in CP-0 (Fig. 4i), hence the CP composite strategy promotes the anisotropic Jahn–Teller distortion to improve the structural stability of γ-MnO_2_.

### In Vitro Cell Toxicity

**Fig. 5:**
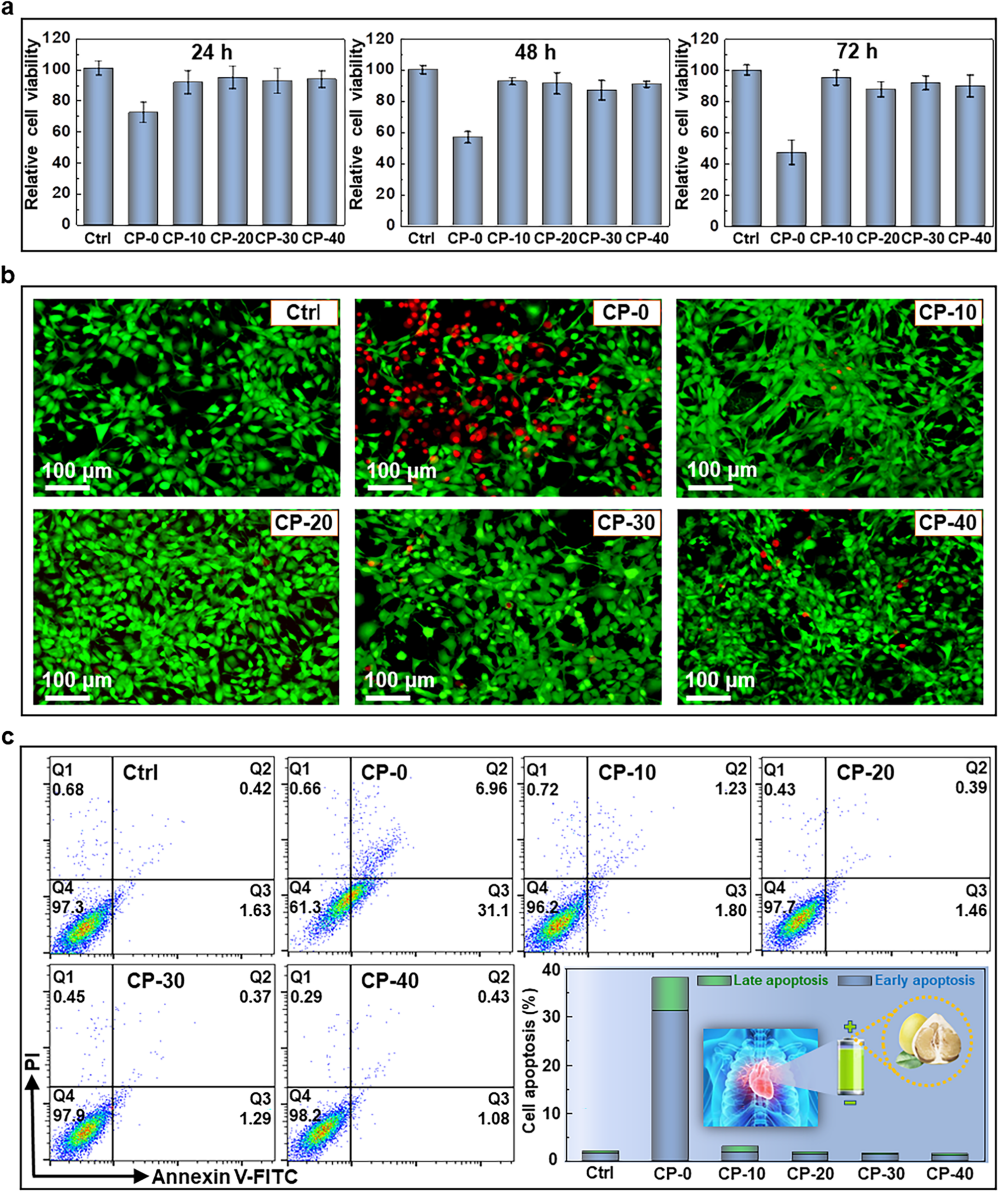
** a** Relative cell viability of 3T3 cells at different time points post treatment. **b** Fluorescence microscopic images of 3T3 cells by Calcein AM/PI staining at 48 h with various treatments. **c** Cell apoptosis percentage of 3T3 cells upon different treatments for 48 h by flow cytometry (Ctrl: cells treated with PBS)

To investigate the intrinsic cytotoxicity of the above-mentioned samples against 3T3 cell, CCK-8 assay is performed to determine the relative cell viability after 24, 48, and 72 h of incubation, respectively. Currently, cells treated with PBS are set as a reference. Negligible proliferation inhibition is observed in cells with the treatment of CP-10, CP-20, CP-30, and CP-40 even up to 72 h of incubation compared with the control group, while exposure to CP-0 exhibits notable cytotoxicity to cells as the relative cell viability decreases over 50% after 72 h (Fig. 5a), indicating the desirable biocompatibility of the above samples except CP-0. Besides, Calcein AM/PI staining is carried out to assess the live/dead cell after different treatments, and no evident cell death is noticed in cells under these above treatments other than CP-0 as expected (Fig. 5b), which is consistent with the results of CCK-8 assay. Furthermore, flow cytometry is performed to investigate the apoptosis profile of 3T3 cells incubated with each sample for 48 h, and no apoptosis is induced by most of the as-synthesized materials with a total apoptotic cell rate less than 5% compared to the control group, but exposure to CP-0 results in severe cell apoptosis as indicated by nearly 40% of total apoptosis percentage (Fig. 5c), and it is in good accordance with the cell toxicity results. It is verified that CP-10, CP-20, CP-30, and CP-40 possess better biosafety compared with CP-0, which might be attributed to the loading of biocompatible CP. Therefore, the CP composite strategy shows a great potential not only in the field of large-scale energy storage, but also in clinical applications.

## Conclusion

The environmental friendly AZIBs with considerable theoretical capacity (820 mAh g^−1^) and appropriate redox potential (− 0.763 V versus standard hydrogen electrode) attract researchers’ broad concern recently [109–117]. Thereinto, biocompatible AZIBs are proposed as candidates for powering biocompatible electronics due to their excellent features of low cost, high-level safety and high-performance [118–120]. Hence, the biomass CP derived from waste grapefruit peel is successfully prepared, and the electrochemical properties and biocompatibility for the composite cathode of γ-MnO_2_ loaded on CP are simultaneously investigated in the present work. The considerable electrochemical properties of 3000 times long cycle stability at 5 A g^−1^ (92.17%), energy density (553.12 Wh kg^−1^), and coulombic efficiency (~ 100%) for the composite cathode with CP quality percentage of 20 wt% are achieved, which is mainly ascribed to the effective regulation of Mn–O bond distance, Mn valence, and Mn domains combined with experimental and DFT computational analysis. Furthermore, the cathodic biosafety is also verified via in vitro test extensively. In brief, this work not only brings forward a feasible countermeasure for structural regulation of multi-function Mn-based cathode with inexpensive biomass-derived carbon, but also paves a novel way for the application of AZIBs in biomedical field.

## Supplementary Information

Below is the link to the electronic supplementary material.

Supplementary file 1 (PDF 946 kb)
