# Supplementary material for: Discovering Cathodic Biocompatibility for Aqueous Zn–MnO2 Battery: An Integrating Biomass Carbon Strategy
Source: Nanomicro Lett. 2024 Feb 5;16:109. doi: 10.1007/s40820-024-01334-3 (PMC10844190; doi:10.1007/s40820-024-01334-3)
Supplement: Supplementary file 1 — (PDF 946 kb) [file 40820_2024_1334_MOESM1_ESM.pdf]

Supporting Information for

Discovering Cathodic Biocompatibility for Aqueous Zn-MnO_2 Battery: An Integrating Biomass Carbon Strategy

Wei Lv[1,*], Zilei Shen[1], Xudong Li[1], Jingwen Meng[1], Weijie Yang[2], Fang Ding[3,*], Xing Ju[1], Feng Ye[1], Yiming Li[4], Xuefeng Lyu[1], Miaomiao Wang[1], Yonglan Tian[1], Chao Xu[1,*]

[1] Institute of Energy Power Innovation, North China Electric Power University, Beijing 102206, People's Republic of China

[2]Department of Power Engineering, School of Energy, Power and Mechanical Engineering, North China Electric Power University, Baoding 071003, People's Republic of China

[3]Key Laboratory of RNA Biology, Institute of Biophysics, Chinese Academy of Sciences, Beijing 100101, People's Republic of China

[4] Collaborative Innovation Center of Integrated Exploitation of Bayan Obo Multi-Metal Resources, Inner Mongolia University of Science and Technology, Baotou 014010, People's Republic of China

*Corresponding authors. E-mail: luidavid@126.com (Wei Lv); dingfang0707@ibp.ac.cn (Fang Ding); mechxu@ncepu.edu.cn (Chao Xu)

Supplementary Figures

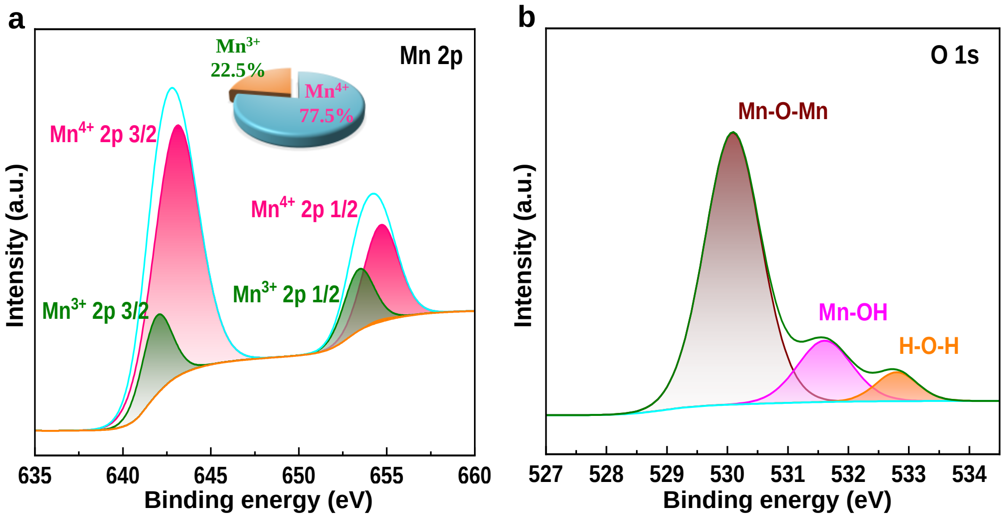

Fig. S1 XPS high-resolution patterns about **a** Mn 2p and **b** O 1s of CP-0

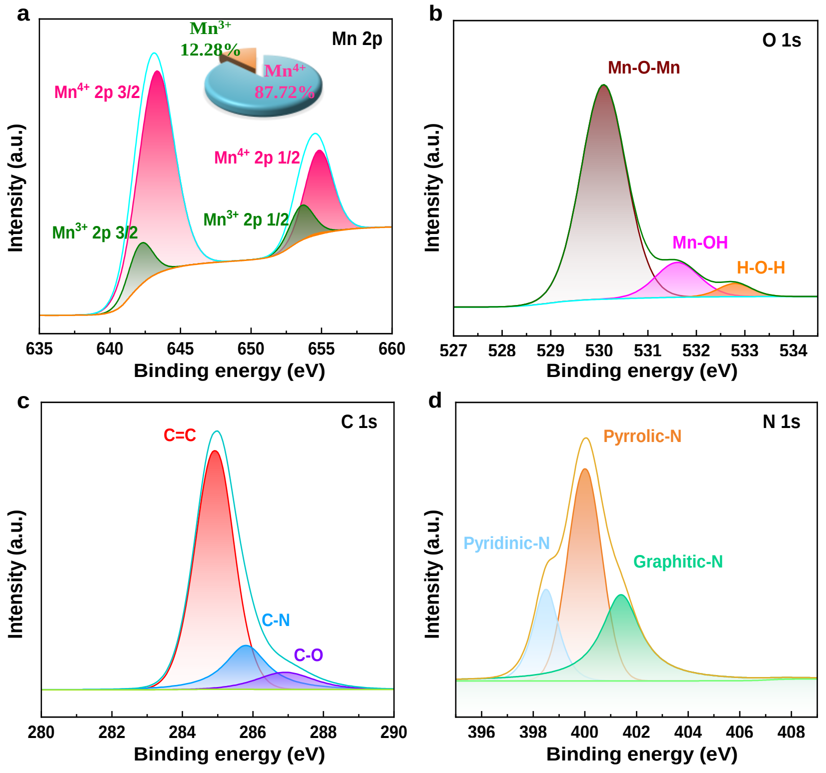

Fig. S2 XPS high-resolution patterns about **a** Mn 2p, **b** O 1s, **c** C 1s, and **d** N 1s of CP-10

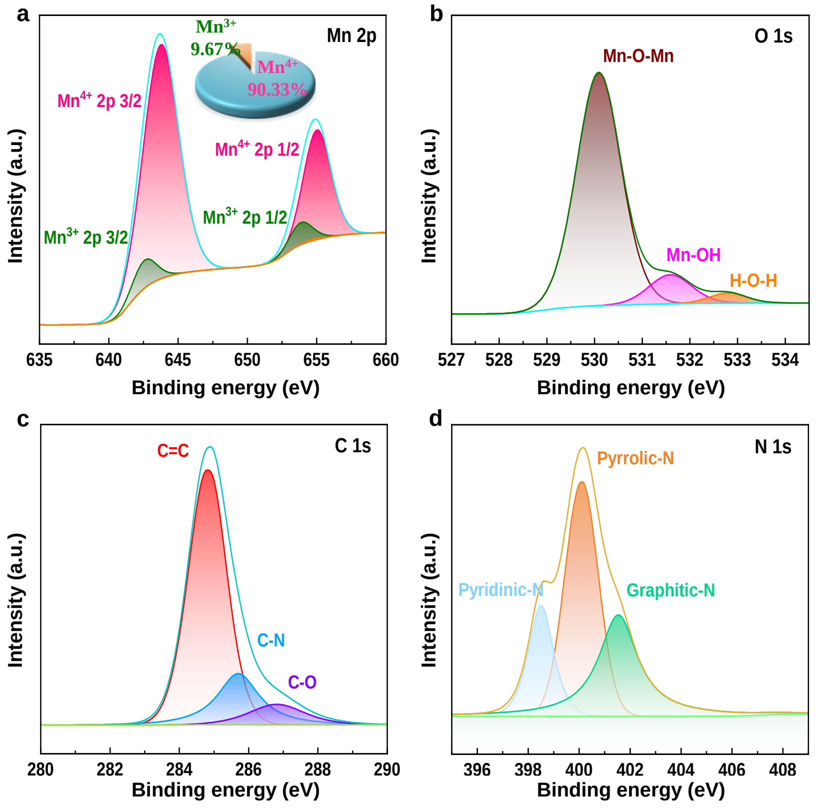

Fig. S3 XPS high-resolution patterns about **a** Mn 2p, **b** O 1s, **c** C 1s, and **d** N 1s of CP-30

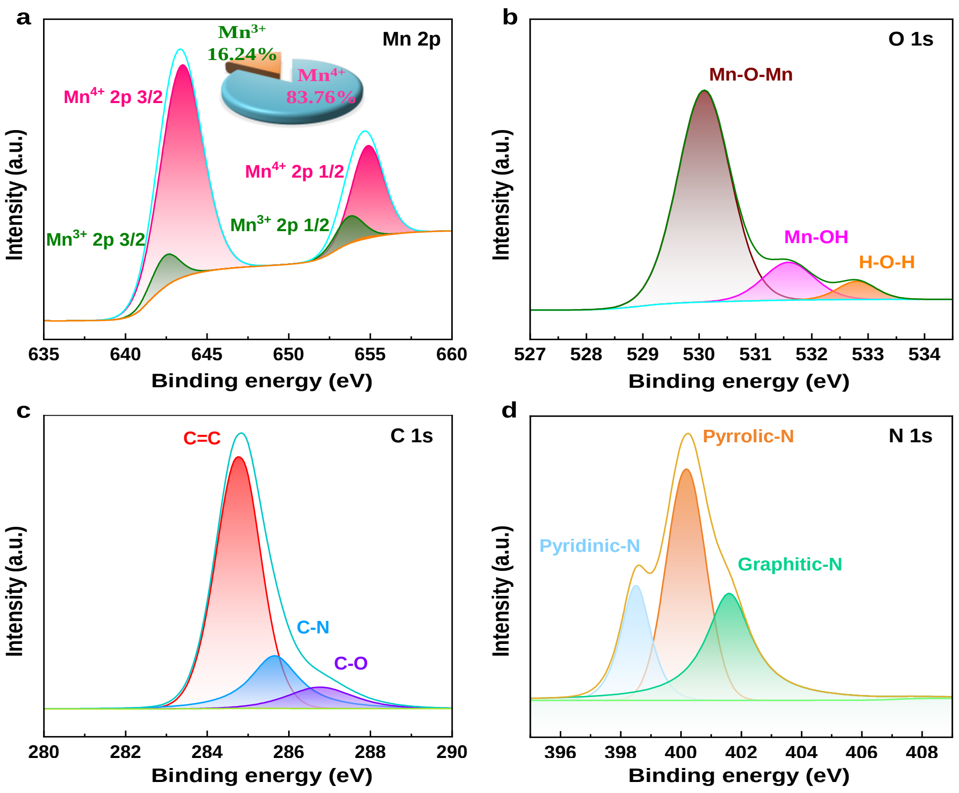

Fig. S4 XPS high-resolution patterns about **a** Mn 2p, **b** O 1s, **c** C1s, and **d** N1s of CP-40

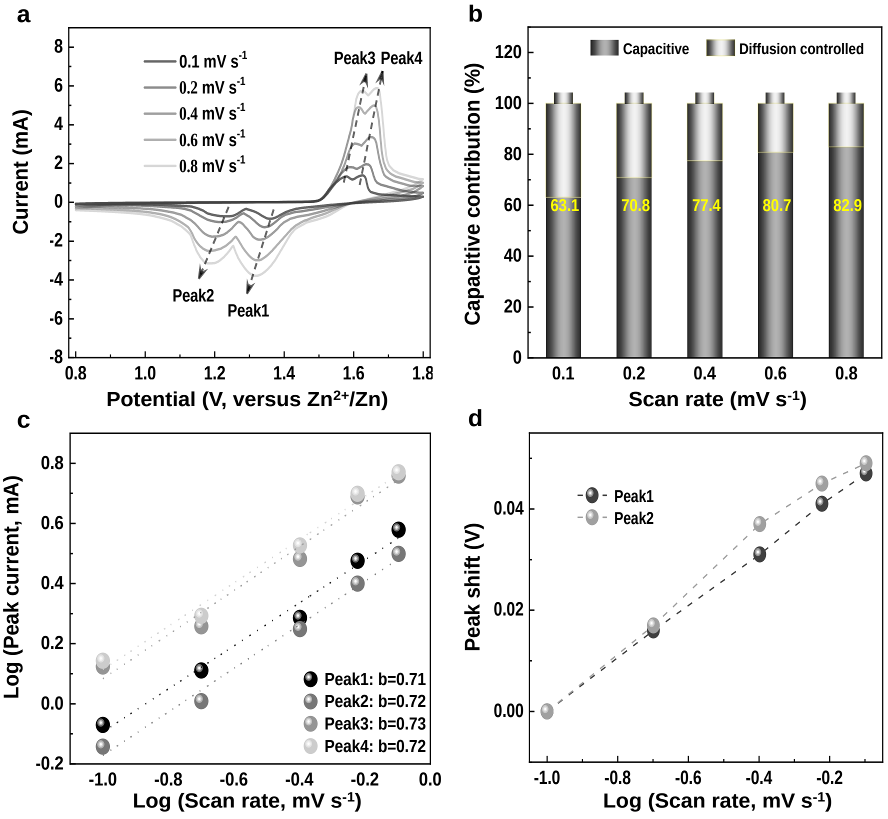

Fig. S5 The **a** CV curves, **b** pseudocapacitive contribution, **c** *b*-value fitting results, and **d** cathodic peak1 and peak2 variation at different scan rate of CP-0

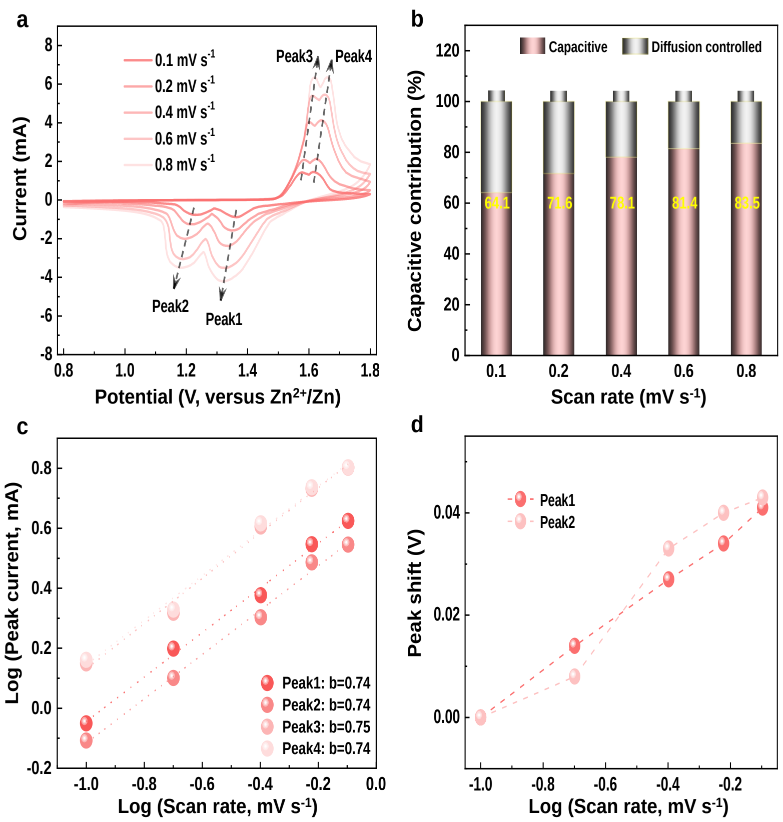

Fig. S6 **a** CV curves, **b** pseudocapacitive contribution, **c** *b*-value fitting results, and **d** cathodic peak1 and peak2 variation at different scan rate of CP-10

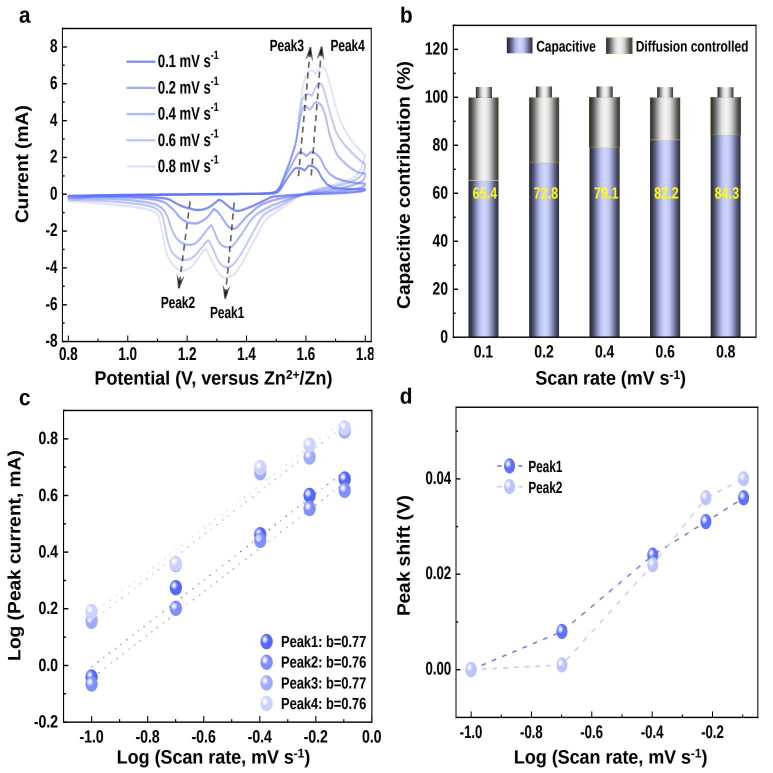

Fig. S7 The **a** CV curves, **b** pseudocapacitive contribution, **c** *b*-value fitting results, and **d** cathodic peak1 and peak2 variation at different scan rate of CP-20

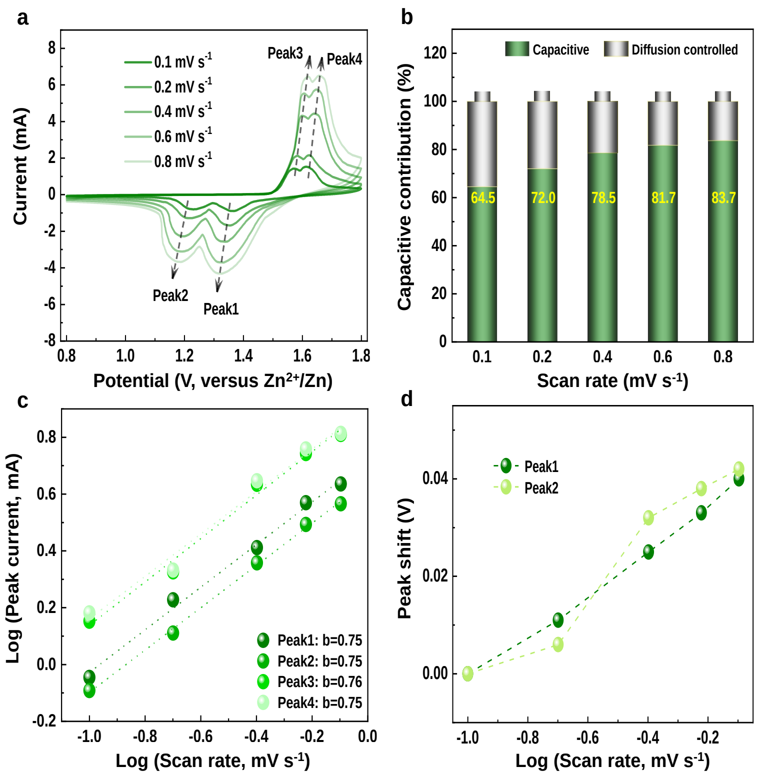

Fig. S8 a CV curves, **b** pseudocapacitive contribution, **c** *b*-value fitting results, and **d** cathodic peak1 and peak2 variation at different scan rate of CP-30

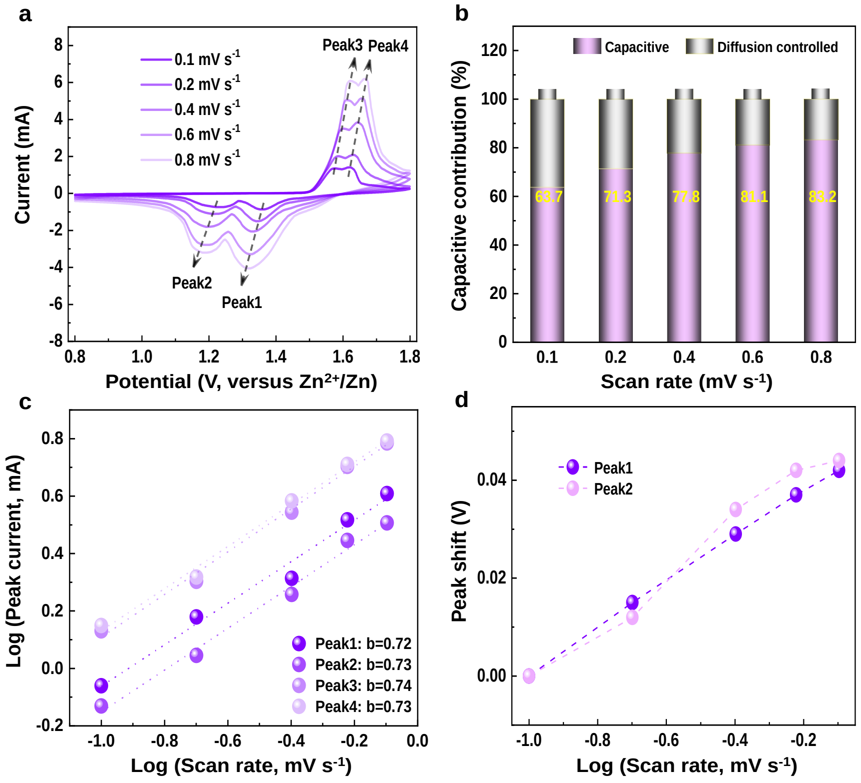

Fig. S9 a CV curves, **b** pseudocapacitive contribution, **c** *b*-value fitting results, and **d** cathodic peak1 and peak2 variation at different scan rate of CP-40 at different scan rate of CP-40
